# The Fibrinolytic System: Mysteries and Opportunities

**DOI:** 10.1097/HS9.0000000000000570

**Published:** 2021-06-01

**Authors:** Robert L. Medcalf, Charithani B. Keragala

**Affiliations:** 1 Molecular Neurotrauma and Haemostasis, Australian Centre for Blood Diseases, Central Clinical School, Monash University, Victoria, Australia

## Abstract

The deposition and removal of fibrin has been the primary role of coagulation and fibrinolysis, respectively. There is also little doubt that these 2 enzyme cascades influence each other given they share the same serine protease family ancestry and changes to 1 arm of the hemostatic pathway would influence the other. The fibrinolytic system in particular has also been known for its capacity to clear various non-fibrin proteins and to activate other enzyme systems, including complement and the contact pathway. Furthermore, it can also convert a number of growth factors into their mature, active forms. More recent findings have extended the reach of this system even further. Here we will review some of these developments and also provide an account of the influence of individual players of the fibrinolytic (plasminogen activating) pathway in relation to physiological and pathophysiological events, including aging and metabolism.

## Introduction

“Fibrinolysis” literally refers to the proteolytic removal of fibrin. That fibrin was the first recognized target for this process, gave credence to its name, and drove scientists to understand how this enzymatic process worked. After all, if the fibrin destroying protease(s) could be identified and harnessed, there would be clinical opportunities to bolster this process for therapeutic advantage. The main components of this system were slowly identified yet additional modifiers of the system continue to be revealed in more recent times (below).

The fibrinolytic system is now appreciated for being a highly orchestrated, purpose-built process that ultimately results in the conversion of the abundant plasma zymogenic protein, plasminogen, into its active proteolytic form plasmin.^1^ The 2 classical enzymes engaged with the task of converting plasminogen into plasmin are tissue-type and urokinase-type plasminogen activator (tPA and uPA, respectively). Other proteases can also perform this function (ie, kallikrein^2^), but not as well as tPA or uPA. These enzymes, and plasmin itself, are members of the serine protease family. These enzymes are all subject to tight modulation, an important and necessary feature to limit the magnitude and duration of plasmin’s activity. Indeed when all checkpoints are in place (ie, under normal conditions), the plasma half-life of plasmin was reported to be between 25 and 50 milliseconds.^3^

The primary inhibitors of tPA and uPA, as well as the main inhibitor of plasmin itself, are also grouped into a family of serine-protease inhibitors (the “SERPINS”). Key among these are plasminogen activator inhibitor (PAI)-1 and PAI-2 that act on both tPA and uPA while alpha_2_-antiplasmin has the crucial job of controlling plasmin.^1^ This regulatory effect does not stop with these serpins, as the fibrinolytic system has additional layers of regulation that influence the ability of these proteins to bind to their target substrates. Thrombin activatable fibrinolytic inhibitor (TAFI) is a prime example of this and plays an important role in limiting the ability of plasminogen to assemble itself on the fibrin surface.^4,5^ Fibrin contains exposed lysine residues that provide important docking sites for plasminogen that conveniently harbors 4 lysine binding sites within its kringle domains.^6^ TAFI, a carboxypeptidase, is able to remove the exposed lysine residues from the fibrin surface, thereby discouraging plasminogen away from fibrin, and sparing fibrin from proteolysis. While the fibrinolytic system can also be primed by other co-factors and inducers (eg, heparin, DNA, histones^7,8^), plasminogen can also be targeted to cell-surface receptors (of which there are at least 12^9^). Most of these receptors also contain exposed lysine residues (akin to fibrin). Once plasminogen is bound to the surface of a given cell, plasmin can be formed locally. In this scenario however, the plasmin formed is not necessarily looking for fibrin to cleave; instead it has additional functions, ranging from the promotion of cell migration^10^ to the initiation of intracellular signaling and cytokine release.^11^

Here we will review the current use or manipulation of the fibrinolytic system in clinical medicine, then outline other processes that are impacted by the same process, which is beginning to raise speculation as to whether the current indications for the use of pro- or anti-fibrinolytic agents can be reconsidered. This review will also provide an overview of how the individual regulatory molecules of the fibrinolytic system can perform additional functions, some of which are now being subjected to therapeutic targeting for purposes unrelated to fibrin removal.

### Clinical medicine and fibrinolysis: current use and recent developments

For decades, the fibrinolytic system has been modulated for therapeutic benefit. This is well-cemented in medicine. Thrombolysis for example, is still mainstream in patients with thromboembolic conditions (ischemic stroke, pulmonary embolism, myocardial infarction and even ischemic limbs) albeit under strict guidelines. On the other hand, anti-fibrinolytic agents, most commonly the lysine analogue, tranexamic acid (TXA), are in widespread use to stop bleeding by protecting fibrin-rich blood clots from premature removal by plasmin.

#### Thrombolysis

Streptokinase, derived from the broth of hemolytic streptococci, was the first fibrinolytic agent evaluated in humans in the 1940s.^12^ This was prior to the discovery and development of either tPA or uPA that were both subject to clinical development initially in the 1950s, but became prominent in the 1980s when recombinant DNA technology permitted large scale protein production. Thrombolysis was initially used for patients with myocardial infarction, but in the mid-1990s, tPA was specifically approved for patients with acute ischemic stroke, albeit with limitations.^13^ tPA is still the more widely used thrombolytic due to the fact that it, unlike uPA, binds preferentially to fibrin, and markedly potentiates (~500-fold) the ability of tPA to activate plasminogen.^14^ Fibrin therefore initiates its own demise^15^; an essential requirement given that it is only formed as a temporary matrix. Even today, thrombolysis using tPA (alteplase) remains the front-line treatment for patients with acute ischemic stroke, despite the advent of endovascular clot retrieval (ECR).^16^

One of the short-comings of tPA is its short plasma half-life of ~5 minutes.^17^ This posed a drug delivery challenge in emergency departments as a 100% bolus administration of tPA would not last long enough to perform its lytic function. In order to maintain a lytic condition for long enough to remove any offending blood clot, tPA needs to be delivered as a 10% bolus followed by a 1 hour infusion. It was realized early on that a thrombolytic with an extended plasma half-life would be far more practical, particularly when dealing with such time sensitive conditions as an acute ischemic stroke where the earlier the administration of thrombolysis, the better. This practical consideration was the driving force behind the development of third-generation thrombolytic agents.^18^ These variants were engineered to create a tPA-like molecule with a longer plasma half-life without compromising any of its other beneficial features. tPA is a complicated multi-domain molecule that contains 2 kringle domains, an epidermal growth factor domain and a finger domain, in addition to its critically important protease domain.^19^ These other domains provide the means for tPA to participate in a myriad of “extra-curricular” functions^20^ (and see below). However, in the context of conventional fibrinolysis and thrombolysis, the key feature to preserve was its remarkable ability to selectively bind to fibrin and less so to fibrinogen. The most successful of these variants generated was tenecteplase (TNK), developed in 1994.^21^ TNK has only 6 amino acids different from tPA and shares 97% amino acid identity with tPA. Nonetheless, these substitutions empowered TNK with an increase in plasma half-life to 30 minutes, a simple change that has been warmly welcomed in the clinical arena. Fibrin selectivity was not only maintained in TNK, but actually enhanced (8-fold), thereby providing a longer lasting tPA-like molecule with even more preference for fibrin.^21^

While ECR has certainly made its impact on clinical management and outcome for patients with ischemic stroke, it is restricted to patients with large vessel occlusions and requires sophisticated infrastructure. Also, ECR usually occurs in conjunction with thrombolysis rather than instead of it. Although it is hard to imagine that there will ever be a more potent fibrinolytic enzyme than plasmin itself, more ingenious approaches might be forthcoming that are more efficient at generating plasmin, producing it in a more targeted manner, ideally only at the clot surface.

#### Natural variation in fibrinolytic capacity

Notwithstanding the above discussion about the current use of thrombolytics, there is an assumption that administration of a given thrombolytic will result in a comparable ability to generate plasmin in any individual. Challenging this notion is a study from 1992 by Tait et al,^22^ who compared the capacity of plasma from over 9000 apparently healthy individuals to generate plasmin ex vivo after the addition of a fibrinolytic agent (in this case streptokinase). The approach was simply to use the plasmin sensitive substrate S2251 to measure the rate of plasmin generation in plasma after the addition of streptokinase. It was revealed in this study that the rate of plasmin generation varied over an 8-fold range, and was further influenced by age, sex, and use of the contraceptive pill.^22^ This is a very important finding when considering the fact that the use of thrombolytic agents in patients with acute ischemic stroke fails to improve outcome in >60% of cases.^23^ While this has been attributed to clot burden or stroke severity, it is possible that the degree of plasmin generation following thrombolysis is simply not the same in all patients and may contribute to this high fail rate.^24^ Some individuals may simply be less capable of responding to tPA to the extent required for thrombolysis.

#### Anti-fibrinolytics

The anti-fibrinolytic agent TXA was developed in Japan in the early 1960s^25^ and is still used widely to reduce bleeding in various conditions, including trauma, post-partum hemorrhage, hemophilia, and also prophylactically in some surgeries. Being a lysine analogue and not a direct plasmin inhibitor (ie, like aprotinin), only lysine-dependent plasmin generation will be blocked by TXA, while plasmin formed through lysine-independent means will be spared from inhibition. This may be one reason why TXA is a safe drug since it cannot completely inhibit plasmin formation. Nonetheless, some have argued that TXA might indirectly promote thrombosis, given that it shuts down the fibrinolytic process, which is lysine-dependent. However, most large scale clinical trials and other meta-analyses have not raised any safety concerns.^26^ On the other hand, the recent “Effects of a high-dose 24-hour infusion of tranexamic acid on death and thromboembolic events in patients with acute gastrointestinal bleeding” trial that evaluated TXA in patients with upper and lower gastrointestinal bleeding showed that thrombosis occurred more frequently in TXA-treated patients.^27^ The reason for why TXA increased thrombotic events in these particular patients is not clear.

### What lies ahead?

The common use of the term “fibrinolysis” does little for the imagination in considering that this system performs anything else other than fibrin removal. The classical view of fibrinolysis is certainly very important and it is not the purpose of this review to downplay this, but rather to highlight the fact that the end-product of this enzymatic cascade, plasmin, has important additional roles in physiology. This functional diversity is not solely related to the pleiotropic effects of plasmin itself (although this is substantial), but that all key modulators of the plasminogen activating pathway, including tPA, uPA, PAI-1, antiplasmin and PAI-2, participate in many other areas of physiology. Some of these processes are independent and unrelated to the activation or inhibition of plasmin. When considering these variables, the clinical manipulation of fibrinolysis takes on a new light where it can no longer be assumed that administration of a thrombolytic agent like tPA for example, *only* activates plasminogen while the administration of TXA *only* protects fibrin.

#### Diversity in function: plasmin

The enzymatic activity initially found in some strains of streptococci that caused fibrin breakdown were first named “fibrinolysin” to reflect fibrin as the substrate. However, after further studies in the 1940s, it was revealed that this moiety did not only cleave fibrin, but also gelatin and casein.^28^ So even back in the 1940s, there was direct evidence that the key fibrin-busting enzyme had other targets. It was because of this non-specificity that the term “fibrinolysin” was renamed “plasmin”, reflecting its activation from its zymogenic precursor, plasminogen, rather than focusing its substrate specificity solely to fibrin.

Plasmin is well known to cleave numerous substrates. But, why produce plasminogen activators that are themselves relatively specific for plasminogen, while the resulting end-product (plasmin) has such broad specificity? Perhaps the key to understanding this (at least in part) stems from our understanding of the process that initiates plasmin generation on the fibrin surface: namely the critical role of lysine residues and how these residues initiate the entire process thereby providing a form of “specificity”. Once lysine residues become visible to plasminogen, plasminogen binds to fibrin and plasmin is formed exactly at the site needed. However, could the broad specificity of plasmin allow similarly targeted proteolysis to occur on different substrates? In this context, it is important to mention that plasminogen was found to bind to dead cells ~100-fold more than to live cells.^29^ Samson et al^30^ subsequently showed that protein aggregates/necrotic tissue formed in necrotic cells provided a non-fibrin cofactor for plasminogen activation that resulted in removal of these aggregates/necrotic material. What was important here was that plasminogen was also recognizing lysine residues formed in these structures as this process was blocked by the lysine analogue TXA. In other words, the removal of both fibrin and misfolded/aggregate proteins were being initiated by the exact same process: the presence of exposed lysine residues that attracts plasminogen to the fibrin or necrotic cell surfaces, where plasmin can be generated to remove the protein in question. Hence, the in vivo function of the fibrinolytic system also includes the removal of misfolded proteins and is therefore important in maintaining protein homeostasis. While it has been well known that plasminogen-deficient mice display increased fibrin deposition, these mice also have increased levels of misfolded proteins after traumatic brain injury.^31^ Adding further intrigue to this effect, plasmin formation not only facilitated the proteolytic removal of these proteins, but also enhanced phagocytosis of macrophages and dendritic cells,^32,33^ while at the same time suppressing the immune response.^32^ This immunosuppressive effect was speculated to minimize self-reactivity that might occur to misfolded proteins.

The number of substrates that plasmin acts upon is quite extensive with many linked with hemostasis including von Willebrand factor,^34^ and many of the coagulation factors (F), including FV, FVII, FIX, FX (see Ref. 35, 36), protease activated receptor (PAR)-2,^37^ as well as the contact pathway components (FXII and pre-kallikrein; see Ref. 38). Plasmin has also been reported to convert FX from a coagulation zymogen into a fibrinolysis cofactor.^39^

What is also worth mentioning is that plasmin also acts to initiate other enzymatic or biological processes. Key among these include plasmin’s ability to activate a number of the matrix metalloproteinases (MMPs^40^), brain-derived neurotropic factor^41^ and transforming growth factor β, and key complement proteins (C3 and C5^42^) into their respective mature forms. Plasmin can also activate signaling pathways downstream of any one of the 12 plasminogen receptors that exist on various leukocytes.^9^

Plasmin formation, mostly in response to tPA, has also been shown to be involved in wound healing,^43^ and with many functions in the brain including memory formation^44^ and in the addictive response to alcohol,^45^ morphine,^46^ methamphetamine,^47^ cocaine,^48^ and nicotine.^49^ These effects are most likely related to the capacity of tPA/plasmin to engage in synaptic plasticity that in some way enhances the addictive response. Consistent with this, overexpression of tPA in the nucleus accumbens (the area of the central nervous system [CNS] important in the addictive response) potentiated sensitivity to many of these addictive agents.^50^ Although this was not formally shown to be plasmin-mediated, the fact that plasminogen-deficient mice also displayed resistance to some of these additive agents makes this highly likely.

#### Diversity of function of the other key components of the fibrinolytic cascade

It comes without surprise that the major components of the fibrinolytic system also participate in processes unrelated to plasminogen activation, and also result in plasmin generation for purposes other than fibrin removal. The following sections will present some examples of the non-canonical functions of these proteins.

#### Tissue-type plasminogen activator

The importance of tPA in activating plasminogen is without doubt. However, tPA does cleave other substrates and has the capacity to bind to cell surface receptors and to promote cell signaling. One example of a non-plasminogen substrate for tPA was revealed by a Swedish group in the mid-2000s, where tPA was shown to directly activate (ie, independent of plasminogen) the platelet-derived growth factor (PDGF)-CC molecule.^51^ This in turn allowed PDGF-CC to bind to its cognate receptor (PDGFRα) and to initiate a protein tyrosine kinase-dependent intracellular signaling event. Indeed, activation of this pathway resulted in opening of the blood-brain barrier (BBB) that occurred following tPA treatment in a mouse model of ischemic stroke. Moreover, this BBB opening event was blocked using the tyrosine kinase inhibitor, imatinib.^52^ It is of immense interest that imatinib is currently being evaluated in a randomized clinical trial of patients with acute ischemic stroke to see if it reduces the deleterious effect of tPA at promoting symptomatic intracranial hemorrhage.^53^

tPA can also interact with cell surface receptors, including the low-density lipoprotein receptors, notably low density lipoprotein receptor-related protein 1 (LRP-1)^54,55^. tPA has also been reported to promote various effects in the CNS (see Ref. 56 for review). Many of these studies did not determine whether the said effect was a direct effect of tPA or required plasmin formation. There are, however, some clear examples where some of the CNS effects of tPA are direct. Indeed, tPA was also reported to activate microglial cells and to initiate cytokine release in a manner not only independent of plasmin formation, but entirely independent of its proteolytic activity. In this case, tPA was essentially acting as a ligand and was actually referred to as being a cytokine.^57^ This was also revealed in a later study where an inactive tPA variant, also referred to as a “cytokine”, was able to induce MMP-9 expression in kidney fibroblasts in a manner dependent on binding to LRP-1.^58^ It was also during this period that tPA was shown to be neurotoxic,^59^ an important yet worrying finding given the use of tPA for thrombolysis in patients with ischemic stroke. This process was dependent on the catalytic activity of tPA which also relied on plasmin generation since the damaging effect was lost in plasminogen-deficient mice.^60^ This example is included because other reports suggested that tPA was promoting neurotoxicity by directly cleaving the NR1 subunit of the glutamate (N-methyl-D-aspartate) receptor.^61^ This was a point of much controversy^62^ and could not be replicated by others.^63,64^ Some reports indicated that NR1 cleavage was secondary to plasmin formation,^62,65^ while others reported that tPA was able to directly stimulate neurons without any NR1 cleavage but required other cell surface receptors in this process.^65^

#### Urokinase

uPA is the other major plasminogen activator in mammalian plasma. uPA is more often linked to plasmin-mediated proteolysis in the extravascular compartment whereas tPA is more associated with conventional fibrinolysis (as well as its other attributes). uPA is often inextricably linked to a unique uPA cell surface receptor (uPAR^66^) where it can perform various functions, some linked to cell-surface plasminogen activation and others to intracellular signaling,^67^ particularly in the context of malignancy as recently reviewed.^68^ uPA and uPAR have also been associated with cell adhesion,^69,70^ neointimal formation, and atherosclerosis.^71^

#### The PAIs

The 2 major PAIs (PAI-1 and PAI-2) have also been implicated in other processes. PAI-2 has an additional anomaly being predominantly located intracellularly, although some is secreted and does influence the conventional fibrinolytic process in both thrombus resolution^72^ and cancer metastasis.^73^ PAI-2 is still viewed as being enigmatic^74^ with suggested influence on apoptosis,^75^ human immunodeficiency virus replication,^76^ monocyte proliferation and differentiation,^77^ and more recently, to block tumor growth.^78^ It is also impressively upregulated in response to inflammatory cytokines, including tumor necrosis factor (TNF).^79^ Early reports indicated that PAI-2 levels can increase over 1000-fold in some cells in response to TNF in combination with the phosphatase inhibitor, okadaic acid.^80^ PAI-2 is also highly regulated by aryl hydrocarbon receptor ligands and by implication, with the process of carcinogenesis as recently reviewed.^81^

PAI-1 on the other hand, shares little in common with PAI-2 apart from the fact they both inhibit tPA and uPA. PAI-1 is a particularly interesting serpin and has also been associated with numerous other processes (see Ref. 82). As it has the ability to interact with matrix proteins, including vitronectin,^83^ PAI-1 is linked to tissue remodeling, cell migration, and intracellular signaling, with implications in the development of fibrosis,^84^ obesity,^85,86^ and quite curiously, the process of cellular senescence.^87^ Concerning the latter, a null mutation in the *PAI-1* (*SerpinE1*) gene was reported to increase aging in humans.^88^ This was revealed in an Amish community in the United States that carried a null mutation in the *PAI-1* gene. These individuals had “longer telomere length”, a more favorable metabolic profile with lower prevalence of diabetes. Exactly how PAI-1 imposes these effects on these parameters is not clear but of immense interest. PAI-1 can also modulate Notch signaling with subsequent effects on endocardial proliferation and maturation.^89^ These associations have led investigators to develop specific PAI-1 inhibitors^90–93^ for some indications (ie, fibrosis, obesity, metabolic disorders among others; see ^94^) and results are eagerly awaited. It is also notable that antibodies against TAFI are also being explored,^95^ but more in relation to conventional fibrinolysis.

#### Alpha_2_-antiplasmin

Antiplasmin has received relatively little attention compared to most other members of the fibrinolytic system, but there has been a resurgence of interest in recent years. Deficiency of antiplasmin resulted in the resolution of venous thrombus^96^ and this prompted efforts to therapeutically target antiplasmin in order to increase endogenous fibrinolytic activity.^97^ This approach was successful in a model of pulmonary embolism^97^ and ischemic stroke.^98^ Indeed, antibodies specific to antiplasmin removed pulmonary emboli in a manner similar to exogenous tPA.^97^

Antiplasmin expression has also been detected in the brain hippocampus. Injection of neutralizing antibodies against antiplasmin was reported to increase hippocampal neurogenesis and spatial memory in mice.^99^ Similarly, antiplasmin-deficient mice display impaired motor and cognitive function and show anxiety and depression-like behavior.^100^ Whether this is due to uncontrolled plasmin activity is not clear but likely. Other reports have indicated additional functions for this serpin that appear to be unrelated to plasmin inhibition. For example, early studies suggested that antiplasmin promoted myofibroblast differentiation independent of plasmin inhibition.^101^

**Figure 1.**
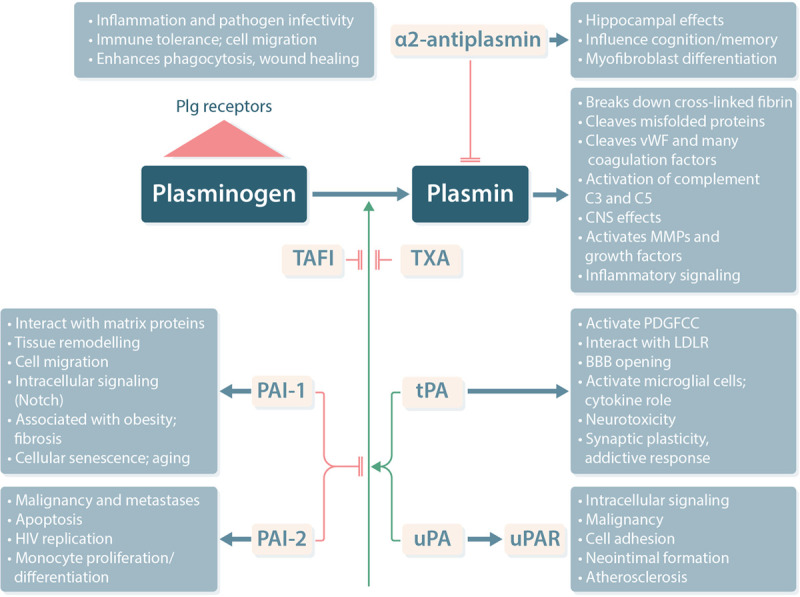
**Schematic representation of the fibrinolytic system and the broad effects of its component parts.** Plasminogen is activated to plasmin by either tPA or uPA and can be inhibited by PAI-1 or PAI-2. Activation can also be endogenously inhibited by TAFI or therapeutically by TXA. Antiplasmin blocks plasmin activity. Additional effects of the individual components of this system are also indicated. Many additional effects of uPA are mediated by its interaction with its receptor, uPAR. Note, this list is not exhaustive. BBB = blood-brain barrier; CNS = central nervous system; HIV = human immunodeficiency virus; LDLR = low-density lipoprotein receptor; MMP = matrix metalloproteinase, PAI = plasminogen activator inhibitor; PDGFCC = platelet derived growth factor-CC; plg = plasminogen; TAFI = thrombin activatable fibrinolysis inhibitor; tPA = tissue type plasminogen activator; TXA = tranexamic acid; uPA = urokinase type plasminogen activator; uPAR = uPA cell surface receptor; vWF = von Willebrand factor.

A schematic representation of the various functions of the fibrinolytic system and the individual components is presented in Figure 1.

#### Unanticipated effects of antifibrinolytic agents

As the broadening role of plasmin has become evident, it is easy to imagine that the use of antifibrinolytic agents might have outcomes unrelated to their intended use, which is simply to stop bleeding. For example, administration of TXA to non-diabetic patients undergoing cardiac surgery resulted in a significant reduction in surgical site infection rates that was independent of its haemostatic effect.^102^ That this was not seen in diabetic patients underscores the confounding effect of diabetes on the fibrinolytic process. TXA was also reported to reduce periprosthetic joint infection after primary arthroplasty^103^ and revision arthroplasty,^104^ although in the 2 examples related to arthroplasty, immune parameters were not evaluated and this effect was attributed to reduction in blood transfusion requirements.

More recent preclinical studies have yielded some surprising effects of TXA in other contexts. For example, TXA was reported to increase life span of immune-compromised mice that coincided with reduced levels of TNF, interleukin-6 and MMP-9.^105^ The same group also reported that long term (2 year) treatment of normal mice with TXA or aprotinin improved cognition and lowered brain amyloid levels.^106^ Whether these longer term effects of TXA are actually related to plasmin inhibition or some other effect remains to be seen.

## Conclusions and future “fibrinolytic” prospects

There is now a growing sentiment that is calling for a reappraisal of “fibrinolysis”. Indeed, there is a lot more to this process than the removal of fibrin, and this is slowly gaining traction in the broader scientific community. Recent reviews have highlighted this very issue.^107^

For decades, fibrinolysis has been largely ignored in comparison to the interest in the parallel coagulation cascade. Minimizing thrombosis risk is effectively tackled using various approaches to slow down the coagulation cascade either by blocking the vitamin K–dependent coagulation enzymes in a general sense (warfarin), accelerating thrombin inactivation (heparin), or by more directly targeting factor X or thrombin (various direct oral anticoagulants). Only when thrombosis really gets out of hand is a direct fibrinolytic approach considered (ie, thrombolysis) and even then, within strict guidelines.

The intrigue of scientists towards coagulation also resulted in advanced laboratory approaches to measure almost every step of the cascade used for diagnostic purposes. Individuals with deficiencies or modifications of any one of the numerous enzymes and regulatory proteins of the coagulation system were revealed permitting targeted, personalized treatment. Uncertainties can remain and the underlying thrombophilic tendencies in some individuals remains a mystery.

It is not beyond the realms of possibility that a defect in the fibrinolytic pathway may impact on other processes even in the absence of a coagulation anomaly. On the other hand, if there was a deficiency in plasminogen, or if its potential to be activated is impaired, then individuals with either low or dysfunctional plasminogen would be expected to present with a thrombotic phenotype. However, conditions associated with plasminogen deficiency—despite their ultra-rarity (1.6 per million^108^)—do not present with thrombosis, but rather the accumulation of fibrin deposits elsewhere, most commonly on the inner eyelid causing ligneous conjunctivitis. It should be remembered that plasminogen levels in these individuals are low, but not zero so it is possible that the remaining plasminogen was enough to provide sufficient fibrinolytic activity in blood vessels.

The fibrinolytic system is continuing to deliver surprises and has come a long way since its initial association with fibrin removal. Although this is arguably still the most relevant role for this system, particularly in the context of thrombosis and bleeding, its looming role in various non-canonical areas cannot be ignored.

## Disclosures

The authors have no conflicts of interest to disclose.

## Sources of funding

RLM receives grant support from the National Health and Medical Research Council (NHMRC) of Australia, grant ID 1156506.
